# Long-life lithium-sulfur batteries with high areal capacity based on coaxial CNTs@TiN-TiO_2_ sponge

**DOI:** 10.1038/s41467-021-24976-y

**Published:** 2021-08-06

**Authors:** Hui Zhang, Luis K. Ono, Guoqing Tong, Yuqiang Liu, Yabing Qi

**Affiliations:** Energy Materials and Surface Sciences Unit (EMSSU), Okinawa Institute of Science and Technology Graduate University (OIST), Okinawa, Japan

**Keywords:** Batteries, Batteries, Nanoscale materials

## Abstract

Rational design of heterostructures opens up new opportunities as an ideal catalyst system for lithium polysulfides conversion in lithium-sulfur battery. However, its traditional fabrication process is complex, which makes it difficult to reasonably control the content and distribution of each component. In this work, to rationally design the heterostructure, the atomic layer deposition is utilized to hybridize the TiO_2_-TiN heterostructure with the three-dimensional carbon nanotube sponge. Through optimizing the deposited thickness of TiO_2_ and TiN layers and adopting the annealing post-treatment, the derived coaxial sponge with uniform TiN-TiO_2_ heterostructure exhibits the best catalytic ability. The corresponding lithium-sulfur battery shows enhanced electrochemical performance with high specific capacity of 1289 mAh g^−1^ at 1 C and capacity retention of 85% after 500 cycles at 2 C. Furthermore, benefiting from the highly porous structure and interconnected conductive pathways from the sponge, its areal capacity reaches up to 21.5 mAh cm^−2^.

## Abstract (web summary)

It is challenging to optimize catalytic heterostructures for lithium sulfur (Li-S) batteries. Here, authors prepare nanometer-scale TiN-TiO_2_ heterostructures via atomic layer deposition on carbon nanotube sponge to realize stable Li-S batteries with high areal capacity and improved rate capability.

## Introduction

Attributed to the high theoretical energy density (2600 Wh kg^−^^1^), lithium–sulfur (Li–S) batteries are considered as one of the most promising candidates to meet the ever-increasing demand of high-energy rechargeable batteries^1–6^. However, the shuttling effect of lithium polysulfides that causes fast capacity fading and poor cycle life severely hinders practical applications of Li–S batteries^1–3^. To address this issue, various sulfur host materials including porous nanocarbons (e.g., graphene foam and carbon nanotube (CNT) network) and polar compounds (e.g., noncarbon oxides, sulfides, and nitrides) have been introduced to block the lithium polysulfide (Li_2_S_*x*_, 4 < *x* ≤ 8) shuttling physically and chemically, respectively^7–12^. Although these strategies can protect lithium polysulfides from being dissolved into the electrolyte to a certain extent, the shuttling problem of polysulfides is not completely solved, especially under high sulfur loadings^13^. Recent studies have shown that “dredging” other than “blocking” is a better solution to the problem of lithium polysulfide shuttling^14^. The main reason is that the conversion from lithium polysulfides to Li_2_S_2_/Li_2_S is slow during the discharging process, which will result in a large accumulation of dissolved polysulfides and eventually exceed the blocking capability of sulfur hosts. To efficiently dredge lithium polysulfides, catalysts should be introduced to accelerate the conversion rate between polysulfides and Li_2_S_2_/Li_2_S^15,16^.

An ideal catalyst for lithium polysulfides conversion needs to be integrated with three important characteristics: (1) high electrical conductivity to promote electron and ion transport for the conversion reaction, (2) appropriate adsorption ability to stabilize polysulfides, and (3) catalytic ability to speed up the polysulfide conversion^17^. However, it is difficult to find a simple material that can simultaneously satisfy all three requirements. For example, metal oxides (such as TiO_2_) show strong adsorption capability for polysulfides^18–21^, but their intrinsically low electrical conductivity will impede the polysulfides from participating in the further electrochemical reactions. Similarly, although metal nitrides (such as TiN) exhibit good electrical conductivity^22,23^, their weak affinities with lithium polysulfides cannot guarantee sufficient polysulfide adsorption. Recently, heterostructures that combine the advantage of each component have been introduced as improved catalysts to enhance the Li–S battery performance. For example, in the TiN–TiO_2_ interlayer-based Li–S battery system, attributed to the synergistic effect of high catalytic and lithium polysulfide adsorption ability from TiN and TiO_2_, respectively, Li–S battery achieves relatively high capacity retention of 73% over 2000 cycles at 1 C^24^. The WS_2_–WO_3_ heterostructure traps lithium polysulfides by WO_3_ first and then transfers to WS_2_ to accomplish the conversion to Li_2_S_2_/Li_2_S, realizing the capacity retention of 86.1% after 300 cycles under 0.5 C^17^. However, most of these heterostructures are synthesized by several steps including solvothermal reactions and high-temperature post-treatments. The complex fabrication process makes it difficult to reasonably control and optimize the content and distribution of each component, which play critical roles in the catalytic ability of the heterostructured catalysts.

Here, we utilize the atomic layer deposition (ALD) method to fabricate a coaxial CNTs@TiN–TiO_2_ sponge based on the chemical vapor deposition (CVD)-prepared three-dimensional (3D) freestanding CNT framework. Through controlling the thickness of TiO_2_ and TiN layers at the outer surfaces of CNTs and combining with the annealing post-treatment, the coaxial CNTs@TiN–TiO_2_ sponge derived from the CNTs hybrid with 10 nm of TiN wrapped by 5 nm of TiO_2_ exhibits excellent ability to improve the Li–S battery performance with a high specific capacity of 1431 mAh g^−1^ at 0.2 C and high capacity retention of 85% after 500 cycles at 2 C. The main reason is that the continuous interface within the TiN–TiO_2_ heterostructure makes TiO_2_ adsorb lithium polysulfides first and then readily diffuses the polysulfides to TiN to proceed with the subsequent electrochemical catalysis. Meanwhile, with the synergistic contribution of highly conductive CNTs, TiN efficiently catalyzes the polysulfide conversion to Li_2_S_2_/Li_2_S. Furthermore, the porous structure and interconnected conductive pathways of the 3D CNT sponge help accommodate a large amount of sulfur and guarantee its efficient utilization. As a result, the areal capacity of the Li–S battery based on the coaxial CNTs@TiN–TiO_2_ sponge reaches up to 21.5 mAh cm^−2^, which is much higher than those of the commercialized lithium-ion batteries (4 mAh cm^−2^) and comparable with the recently published Li–S battery systems with the sulfur loadings higher than 8 mg cm^−^^2,^^7,13,23,25–36^.

## Results and discussions

**Fig. 1:**
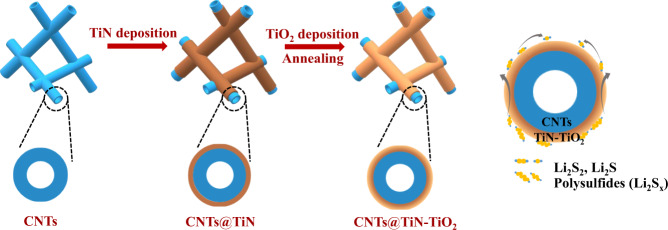
Schematic illustration showing the fabrication process of CNTs@TiN–TiO_2_ and its catalytic process for the polysulfide conversion. Through the ALD method, TiN and TiO_2_ are deposited sequentially on the CNTs surface, and then the obtained hybrid is annealed to be transformed into the coaxial CNTs@TiN-TiO_2_ sponge with uniform distribution of the TiN–TiO_2_ heterostructure. With the help of the TiN–TiO_2_ heterostructure, the conversion process from lithium polysulfides to Li_2_S_2_/Li_2_S occurs smoothly in two steps of adsorption and catalytic conversion.

There are three steps during the fabrication of coaxial CNTs@TiN–TiO_2_ sponge: (1) depositing TiN onto CNTs following the set recipe of ALD (see the Methods for the details) to obtain CNTs@TiN, (2) growing TiO_2_ layer on the outer surfaces of TiN, and (3) annealing the CNT hybrid to promote the uniform distribution of the TiN–TiO_2_ heterostructure, as illustrated in Fig. 1. The 3D porous CNT sponge is an ideal substrate for TiN–TiO_2_ deposition and characterization because of the large number and special tubular structure of multi-walled CNTs, which stack layer by layer to construct the sponge. Specifically, a large number of CNTs (acting as substrates) guarantee abundant material deposition, and the deposited TiN (or TiO_2_) can be identified readily from CNTs by transmission electron microscopy (TEM) without any complex pre-treatment in planar (or micrometer-scale) substrate-based samples, which is beneficial for the structural optimization. Moreover, numerous multi-walled CNTs within the CNT sponge interconnect with one another to provide free pathways for transporting electrons, which circumvents the electron-transport problem in thick powder-form electrodes and shows great advantage in improving the areal capacity of Li–S battery. Different from the commonly used method of loading solid sulfur as the active material, we dropped the lithium polysulfides solution to the CNTs@TiN-TiO_2_ sponge, letting polysulfides soak into the sponge and act as the initial active materials directly. This is mainly for two reasons: (1) solution infiltration is one of the most feasible strategies to load active materials into 3D sulfur hosts uniformly; (2) the matched polarity between TiO_2_ (or TiN) and polysulfides facilitates the efficient stabilization of active materials, which is critical for the cycling stability of Li–S battery. Benefiting from the integrated adsorption and catalytic ability of the TiN–TiO_2_ heterostructure, the loaded lithium polysulfides in the CNTs@TiN–TiO_2_ sponge are stabilized on the hybridized nanotubes first and then smoothly transferred to catalytic TiN to finish the conversion reaction to Li_2_S_2_/Li_2_S as shown in Fig. 1. According to the previous report^37^, the transformation from Li_2_S_2_ to Li_2_S is intrinsically a slow solid–solid reaction process, which can also be accelerated by the catalytic TiN–TiO_2_ heterostructure, improving the utilization of sulfur species.

### Optimization of the TiN–TiO_2_ heterostructure

Attributed to the atomic-scale deposition and intrinsic conformity of ALD, the TiN content can be readily controlled by the deposited thickness on CNTs. Through controlling the deposition cycles, three CNT hybrids with different TiN thicknesses (5, 10, and 20 nm) are fabricated and denoted as CNTs@TiN-5, CNTs@TiN-10, and CNTs@TiN-20. From the TEM results (Fig. S1), the morphology of the CNTs@TiN hybrids, especially the interface between TiN and CNTs is greatly influenced by the deposited TiN thickness. Deposition of a 5 nm-thick TiN layer on the CNT surface can be clearly identified by the low magnification TEM image (Fig. S1a), however, loose deposition on the surface of CNT with some discrete regions (Fig. S1b) is observed under high magnification condition. As the deposited thickness of TiN increases to 10 and 20 nm, the interfaces between CNTs and TiN become continuous and smooth (Fig. S1c–f). This morphology change is mainly attributed to the uneven surfaces of multi-walled CNTs, which impede the atomic deposition of TiN at some defective places, resulting in holes and bumps. To evaluate the electrochemical properties of these three hybrids, the Li–S cells applying them as sulfur hosts are assembled and tested. Although the battery based on CNTs@TiN-5 exhibits the highest specific capacity (about 1300 mAh g^−1^) in the first five cycles among three samples (Fig. S2), CNTs@TiN-10 possesses the best cycling stability with over 1000 mAh g^−1^ after 100 cycles, which is higher than 762 and 712 mAh g^−1^ of CNTs@TiN-5 and CNTs@TiN-20, respectively. By virtue of this cycling stability, it can be concluded that CNTs@TiN-10 with a continuous TiN layer is the optimized structure for the sulfur host. Although CNTs@TiN-20 has a morphology similar to that of CNTs@TiN-10, the electric conductivity measurement results show that the former has worse conductivity for electrons (Table S1), which substantially limits the electron transport and hinders the efficient utilization of polysulfides, resulting in lower specific capacity and inferior cyclic stability. In parallel, the loose and unstable structure of CNTs@TiN-5 is likely to be damaged during the repeated chemical reaction process, causing a fast capacity fading.

**Fig. 2:**
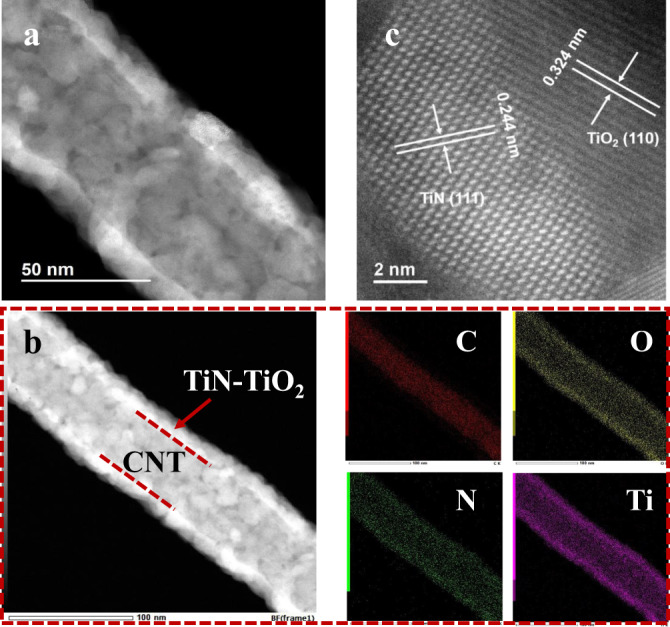
Morphology characterization of CNTs@TiN–TiO_2_-5. **a** TEM image of CNTs@TiN–TiO_2_-5 showing the integrated TiN–TiO_2_ heterostructure coated on the CNT surface. **b** TEM and corresponding elemental mappings of C, O, N, and Ti in CNTs@TiN–TiO_2_-5 showing the mixed and uniform distribution of TiN–TiO_2_ heterostructure. **c** High-resolution TEM of CNTs@TiN–TiO_2_-5 showing the well-matched interface of the TiN–TiO_2_ heterostructure. Source data are provided as a Source Data file.

Based on the above results, CNTs@TiN-10 is regarded as the optimized structure, hence it is applied as the new substrate for TiO_2_ deposition. Similarly, 5 nm of TiO_2_ is grown on the surface of CNTs@TiN-10 by the ALD method to fabricate the coaxial hybrid of CNTs@TiN@TiO_2_. As shown in Fig. S3a, the inner TiN can be readily distinguished from the outer TiO_2_ layer of this hybrid because TiN is much coarser and looser than TiO_2_. However, the Li–S battery performance result shows that the deposited TiO_2_ around the CNTs@TiN severely deteriorates the battery electrochemical performance, especially for the cyclic stability (Fig. S3b). The dense TiO_2_ layer probably blocks the diffusion of polysulfides to TiN and electron transport, which hinders the catalytic conversion of polysulfides to Li_2_S_2_/Li_2_S. In addition, the hybrids directly obtained from the ALD exhibit low crystallinity. From Fig. S4, only one strong peak at around 26° belonging to CNTs is detected in the XRD pattern of CNTs@TiN (10 nm) and CNTs@TiO_2_ (5 nm). Annealing is one of the most efficient post-treatment methods to improve the crystallinity and structures of the materials. To promote the favorable distribution of TiN and TiO_2_, CNTs@TiN@TiO_2_ is annealed within dry N_2_ gas atmosphere. The TEM image shows that the TiN and TiO_2_ layers are mixed to form one integrated layer coated on the CNTs after annealing (Fig. 2) without new crystalline compound formation, which is verified by the XRD pattern of the annealed product (Fig. S5). The amorphous baseline in this XRD result is attributed to the amorphous carbon of the CNT sponge. The main distribution of carbon in the inner part from the corresponding EDX mapping images confirms that CNTs are applied as the original substrate for TiN and TiO_2_ deposition. Interestingly, the elements of titanium, nitrogen, and oxygen wrapping around the CNTs are uniformly presented. It indicates that the annealed outer layer corresponds to a mixture of TiN and TiO_2_, which is consistent with the TEM results. From the high-resolution TEM picture (Fig. 2), the lattice fringes with the spacings of 0.244 and 0.324 nm are indexed to (111) lattice plane of TiN and (110) plane of TiO_2_, respectively. Besides, the TiN–TiO_2_ heterostructure possesses a continuous and atomically matched interface, which is beneficial for the smooth reaction process of polysulfide adsorption, diffusion, and catalytic conversion. For the sake of concise description, the annealed CNTs@TiN@TiO_2_ with TiN-TiO_2_ heterostructure is named as CNTs@TiN–TiO_2_-5, of which the number stands for the thickness of the deposited TiO_2_. Because of the intrinsic conformity of the ALD method, all TiN–TiO_2_ layers are uniformly grown around the outer surface of CNTs, and the hybridized CNTs@TiN–TiO_2_-5 sponge retains its porosity and 3D structure, which is beneficial for high sulfur loadings and efficient electrolyte permeation (Fig. S6). To further optimize the annealed TiN–TiO_2_ heterostructure, two more different thicknesses of TiO_2_ (2 and 10 nm) are deposited and annealed, which are denoted as CNTs@TiN–TiO_2_-2 and CNTs@TiN–TiO_2_-10. Being similar to the CNTs@TiN–TiO_2_-5, CNTs@TiN–TiO_2_-2 has an integrated TiN–TiO_2_ heterostructure layer on the surface of CNTs (Fig. S6). However, a discontinuous and irregular boundary appears in the outer layer of the CNTs@TiN–TiO_2_-10 (Fig. S7). Therefore, it can be concluded that the deposited TiO_2_ thickness (i.e., TiO_2_ content) is an important parameter to influence the TiN–TiO_2_ heterostructure.

### Lithium polysulfide adsorption

**Fig. 3:**
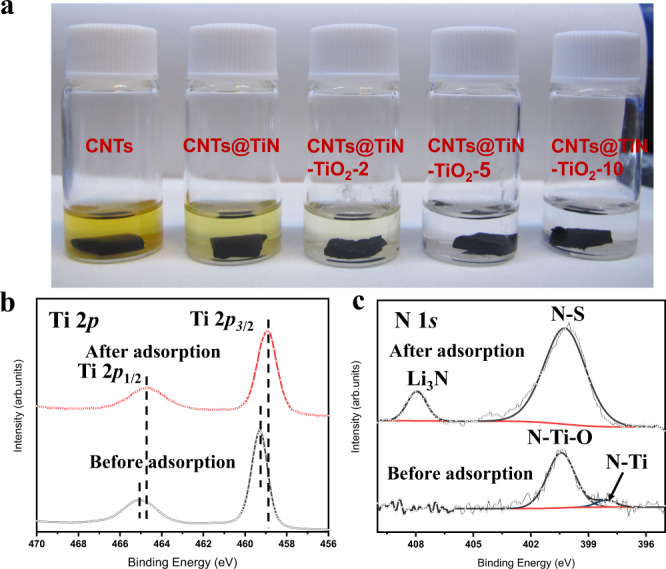
Lithium polysulfide adsorption visual test and corresponding XPS characterization of CNTs@TiN–TiO_2_-5. **a** Comparison of polysulfide adsorption ability of CNTs@TiN–TiO_2_-2, CNTs@TiN–TiO_2_-5, and CNTs@TiN–TiO_2_-10 by immersing these hybrids into the Li_2_S_6_ solution (0.005 M). XPS spectra of **b** Ti 2*p* and **c** N 1*s* in CNTs@TiN–TiO_2_-5 before and after polysulfide adsorption. Source data are provided as a Source Data file.

The catalytic conversion process of lithium polysulfides includes two critical steps of the adsorption and catalytic reaction. To evaluate the adsorption ability of CNTs@TiN–TiO_2_-2, CNTs@TiN–TiO_2_-5, and CNTs@TiN–TiO_2_-10, these three hybrids are dropped into the Li_2_S_6_ solution (0.005 M) and kept overnight (Fig. 3a). The visual test result shows that the sequence of the Li_2_S_6_ adsorption ability is TiO_2_ > TiN > CNTs, which is consistent with the previous results. Besides, with the increase of TiO_2_ content, the polysulfide adsorption ability of the CNT hybrid increases gradually. When the deposited TiO_2_ thickness is 5 nm, the color of the Li_2_S_6_ solution becomes transparent. However, in the Li_2_S_6_ solution with CNTs@TiN–TiO_2_-2, there are still some Li_2_S_6_ residues, which illustrates the limited Li_2_S_6_ adsorption ability of CNTs@TiN–TiO_2_-2 and the importance of the TiO_2_ content optimization (Fig. 3a). There are two main types of adsorption between the host materials and lithium polysulfides, physical adsorption and chemical adsorption. The physical adsorption is closely correlated with the porosity (i.e., specific surface area, pore size, and pore volume) of the materials. From the BET results (Fig. S8 and Table S2), it can be concluded that the values of both specific surface area and mesopore volume increase with the increase of the deposited TiO_2_ amount, which is beneficial for the adsorption of lithium polysulfides. However, because the strength of physical adsorption is always too weak to stabilize polysulfides efficiently, relatively strong chemical interaction in the chemical adsorption has the advantage to trap lithium polysulfides, facilitating the subsequent catalytic conversion reaction. To determine the interaction between the TiN–TiO_2_ heterostructure and lithium polysulfides, X-ray photoelectron spectroscopy (XPS) measurements of CNTs@TiN–TiO_2_-5 before and after adsorption are conducted. Because of the immersion in Li_2_S_6_-contained traditional ether-based electrolyte, there is the appearance of new peaks of fluorine, sulfur, and lithium after adsorption (Fig. S9). As shown in Fig. 3b, the two spin–orbit splitting peaks of Ti 2*p* (Ti 2*p*_1/2_ at 465 eV and Ti 2*p*_3/2_ at 459.4 eV) shift to the positions with lower binding energy (Ti 2*p*_1/2_ at 464.6 eV and Ti 2*p*_3/2_ at 458.9 eV) after Li_2_S_6_ adsorption, which indicates the chemical interactions between Li_2_S_6_ and TiN–TiO_2_ heterostructure. Because of the stronger negativity of sulfur species than Ti, Ti 2*p* tends to accept electrons from polysulfides, resulting in a lower binding energy. The formation of the new peaks of Li_3_N and N–S in N 1*s* core-level region further demonstrates the chemical bonding of lithium polysulfides with the TiN–TiO_2_ heterostructure (Fig. 3c).

### Catalytic ability

**Fig. 4:**
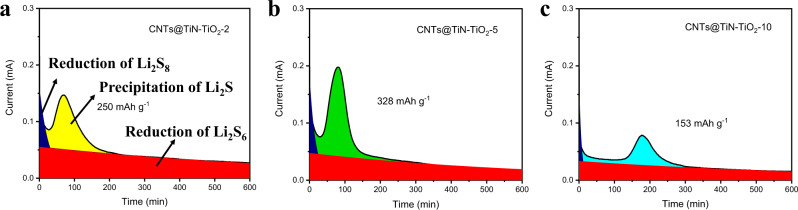
Li_2_S deposition process under potentiostatic discharge condition. Potentiostatic discharge curves of **a** CNTs@TiN–TiO_2_-2, **b** CNTs@TiN–TiO_2_-5, and **c** CNTs@TiN–TiO_2_-10 at 2.05 V. Source data are provided as a Source Data file.

A symmetric cell without the consideration of a lithium metal anode is a common configuration to evaluate the electrochemical kinetics (including the catalytic ability) of sulfur host materials. Utilizing the same material as both the cathode and anode, the symmetric cells of CNTs@TiN–TiO_2_-2, CNTs@TiN-TiO_2_-5 and CNTs@TiN–TiO_2_-10 are assembled and tested by the cyclic voltammetry (CV) method at a scanning speed of 2 mV s^−^^1^. Figure S10 shows that there is not any redox peak when the electrolyte without Li_2_S_6_ is applied in the symmetric cells, which indicates that only Li_2_S_6_ is the active material to carry out the redox reactions in the testing system, excluding the influence from the commonly used ether-based electrolyte. After Li_2_S_6_ is added into the electrolyte, two pairs of redox peaks appear as shown in Fig. S10. Specifically, two anodic peaks correspond to the oxidation of Li_2_S_2_/Li_2_S to lithium polysulfides and further to elemental sulfur (S_8_), and two cathodic peaks are assigned to the reverse reaction process (the reduction of S_8_ to polysulfides and further to Li_2_S_2_/Li_2_S). In CNTs@TiN–TiO_2_-5, these peaks exhibit narrow shapes and their separation is small, illustrating the enhanced lithium polysulfides conversion catalyzed by the TiN–TiO_2_ heterostructure. In contrast, CNTs@TiN–TiO_2_-2 shows broader and wider redox peaks, suggesting the inferior catalytic capability due to the limited adsorption ability for lithium polysulfides. For CNTs@TiN–TiO_2_-10, not only the peaks are severely broadened and widened, but the current intensity is also greatly decreased, indicating the weak catalytic activity of the TiN–TiO_2_ heterostructure with irregular boundaries. These unfavorable defects hinder the diffusion of the polysulfides and therefore deteriorate the catalytic ability. Besides, the inferior electric conductivity induced by the increased TiO_2_ content limits the efficient utilization of lithium polysulfides. It is worth noting that Li_2_S growth is a critical step in the lithium polysulfide conversion process. To investigate the kinetics of Li_2_S precipitation (or growth), the coin cells applying Li_2_S_8_ solution as the electrolyte are first galvanostatically discharged to 2.06 V and then potentiostatically discharged at 2.05 V until the current is lower than 10^−5^ mA. The precipitation current and capacity can be calculated based on the potentiostat discharge curves as shown in Fig. 4 (see the Experimental section for the details). CNTs@TiN–TiO_2_-5 exhibits the highest current (0.2 mA) and capacity (328 mAh g^−^^1^) for Li_2_S precipitation compared to CNTs@TiN–TiO_2_-2 (0.15 mA, 250 mAh g^−^^1^) and CNTs@TiN–TiO_2_-10 (0.75 mA, 153 mAh g^−1^). These results reveal that the CNTs@TiN–TiO_2_-5 possesses the best capability to accelerate the polysulfides conversion reaction (including the Li_2_S precipitation) and promote the efficient utilization of lithium polysulfides.

### Li–S battery performance

**Fig. 5:**
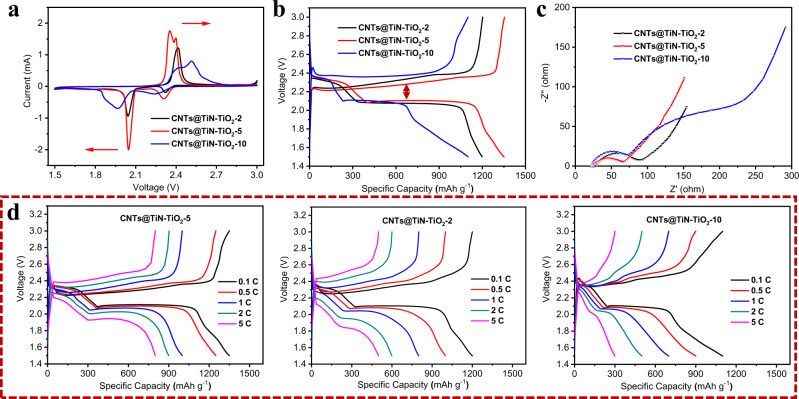
Electrochemical performance of CNTs@TiN–TiO_2_-2, CNTs@TiN–TiO_2_-5, and CNTs@TiN–TiO_2_-10. **a** CV curves at the scan rate of 0.1 mV s^−1^, **b** galvanostatic charge and discharge curves, **c** EIS curves, and **d** rate performance from 0.1 to 5 C of CNTs@TiN–TiO_2_-5, CNTs@TiN–TiO_2_-2, and CNTs@TiN–TiO_2_-10. Source data are provided as a Source Data file.

The electrochemical measurements show that the Li–S battery applying CNTs@TiN–TiO_2_-5 as the sulfur host exhibits the best electrochemical performance including the specific capacity, rate capability, and cyclic stability. From the CV results (the scan rate is 0.1 mV s^−1^) in Fig. 5a, there are two cathodic peaks during the discharge process, corresponding to the reduction of sulfur to lithium polysulfides at higher voltage and the formation of Li_2_S_2_/Li_2_S at a lower voltage, respectively. Besides, two overlapped anodic peaks during the charging process stand for the oxidation of Li_2_S_2_/Li_2_S to lithium polysulfides and elemental sulfur. In CV curves, the separation between the corresponding cathodic and anodic peaks represents the polarization, which is correlated to the electrochemical kinetics of batteries. Theoretically, the smaller polarization reflects better electrochemical kinetics. It can be clearly observed in Fig. 5a that CNTs@TiN–TiO_2_-5 has the sharpest CV peaks, highest current intensity, and smallest polarization in comparison with CNTs@TiN–TiO_2_-2 and CNTs@TiN–TiO_2_-10. Furthermore, CNTs@TiN–TiO_2_-5 exhibits the highest discharge capacity (Fig. 5b). In the galvanostatic charge/discharge curves, the plateaus in discharge and charge curves are attributed to the reduction and oxidation reaction processes of Li–S batteries, which agrees well with the redox peaks in CV curves (Fig. 5b). Similarly, the gap between the discharge and charge curves also stands for the polarization, of which CNTs@TiN–TiO_2_-5 is the smallest among these three hybrids. Charge transfer resistance is an important indicator for the charge (e.g., electrons and lithium ions) transport during the battery working process. The electrochemical impedance spectroscopy (EIS) results show that CNTs@TiN–TiO_2_-5 has the smallest semicircle diameter, which corresponds to the best charge transfer capability and reveals the favorable electrochemical conversion reaction in Li–S battery with CNTs@TiN–TiO_2_-5 as the sulfur host (Fig. 5c). For CNTs@TiN–TiO_2_-10, there are two semicircles with largely increased resistance, which illustrates that the irregular boundary in the hybrid of CNTs@TiN–TiO_2_-10 can severely limit the charge transport and the lithium polysulfide conversion reaction. Benefiting from the favorable electrochemical kinetics, CNTs@TiN-TiO_2_-5 exhibits excellent rate performance. As shown in Fig. 5d, the specific capacities of CNTs@TiN–TiO_2_-5 at the current density of 0.1, 0.5, 1, 2, and 5 C are 1350, 1250, 1000, 900, and 800 mAh g^−1^, respectively. These values are much higher than that of CNTs@TiN–TiO_2_-2 and CNTs@TiN–TiO_2_-10. In addition, CNTs@TiN–TiO_2_-5 possesses the smallest polarization, and the change of the polarization value exhibits the gentlest increasing trend with the increase of the C-rate when compared to the other two hybrids of CNTs@TiN–TiO_2_-2 and CNTs@TiN–TiO_2_-10. It further verifies that the CNTs@TiN–TiO_2_-5 is an ideal host material to promote the polysulfides conversion and improve the electrochemical performance of Li–S batteries.

**Fig. 6:**
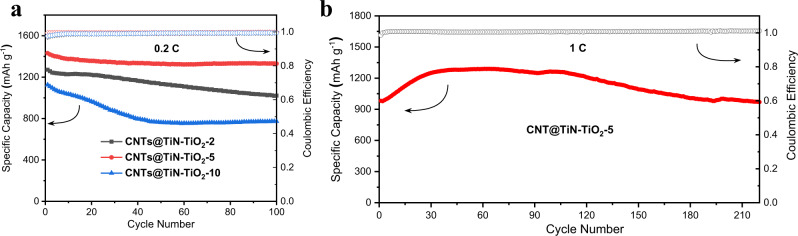
Cycling performance of CNTs@TiN–TiO_2_-2, CNTs@TiN–TiO_2_-5, and CNTs@TiN–TiO_2_-10. **a** Cyclic stability comparison of CNTs@TiN–TiO_2_-2, CNTs@TiN–TiO_2_-5 and CNTs@TiN–TiO_2_-10 after 100 cycles at 0.2 C. **b** Long-term cycling performance of CNTs@TiN–TiO_2_-5 at 1 C. Source data are provided as a Source Data file.

Furthermore, the cycling performance of the Li–S batteries is measured and compared. From Fig. 6, the initial specific capacities of CNTs@TiN–TiO_2_-2, CNTs@TiN–TiO_2_-5 and CNTs@TiN–TiO_2_-10 at the C-rate of 0.2 C are 1271, 1431, and 1125 mAh g^−1^, respectively. After 100 cycles, the capacity of 1330 mAh g^−^^1^ is achieved for CNTs@TiN–TiO_2_-5. In contrast, only 1021 mAh g^−1^ for CNTs@TiN–TiO_2_-2 and 773 mAh g^−1^ for CNTs@TiN–TiO_2_-10 are retained. As the current density increases to 1 C and 2 C, the capacity fading rates are kept at 0.0056% and 0.031% per cycle after 220 and 500 cycles, respectively, which are competitive values for the cyclic performance of Li–S battery compared to other related works (Table S3)^7,13,23,25–36^. Attributed to the 3D structure, the areal sulfur loading of CNTs@TiN–TiO_2_−5 can reach up to 15 mg cm^−2^. Therefore, its highest corresponding areal capacity at 0.2 C is 21.5 mAh cm^−^^2^, which is much higher than the related works focusing on Li–S battery with high areal capacity^25,38–40^. Even at 1 C and 2 C, the highest areal capacity of 19.3 and 12.2 mAh cm^−2^ can be obtained (Figs. S11 and S12). The initial capacity increase during the cycling is possibly due to the activation of high-loading materials under the increased C-rate condition^30,41^. Correspondingly, the energy density based on the whole device is about 269.05 Wh/kg at the C-rate of 0.2 C, which is comparable to the previous works calculated on the whole deive^42,43^.

In summary, we developed a 3D coaxial CNT hybrid coated with a TiN–TiO_2_ heterostructure by the ALD method combining with post-annealing. Through optimizing the deposited TiO_2_ thickness, the ideal heterostructure with the continuous interface can be obtained, which facilitates the smooth process of lithium polysulfide adsorption, diffusion, and catalytic conversion. As a result, the rate performance and cyclic stability of Li–S batteries are enhanced. Furthermore, attributed to the high sulfur loading of the 3D inter-connective network, high areal capacity can be achieved simultaneously. Our strategy might be extended to the optimization of other coaxial/layer-by-layer heterostructures and promote the formation of continuous and well-matched interfaces with promising applications in energy storage and catalysis.

## Methods

### Materials

Nitric acid (HNO_3_, AR) was purchased from Wako. Tetraglyme (99.5%), sulfur (S_8_, 99.9%), and lithium disulfide (Li_2_S, 99.9%) were ordered from Sigma-Aldrich. Tetrakis(dimethylamido)titanium was bought from Japan Advanced Chemicals. All chemicals are analytical grade without further purification.

### Fabrication of CNTs@TiN, CNTs@TiN@TiO_2_, CNTs@TiN–TiO_2_-2, CNTs@TiN–TiO_2_-5, and CNTs@TiN–TiO_2_-10

CNT sponge was synthesized by the CVD method. The catalyst and carbon precursors are ferrocene and 1,2-dichlorobenzene, respectively. Before depositing TiN, the CNT sponge was treated with nitric acid (70% of mass ratio) at 120 °C for 12 h, which was then washed with deionized water until neutral (pH ~7). After being freeze-dried, the CNT sponge was functionalized by carboxylic groups on the outer surfaces of CNTs, which is beneficial for the stable hybridization of sponge with other polar materials (e.g., TiN and TiO_2_). CNTs@TiN and CNTs@TiN@TiO_2_ were fabricated with the recipes at 150 °C by the ALD method in an ALD system (Cambridge Nanotechnology Savannah S200). The precursors for TiN and TiO_2_ depositions are tetrakis(dimethylamido)titanium, and gases of NH_3_ and H_2_O. CNTs@TiN–TiO_2_-2, CNTs@TiN–TiO_2_-5, and CNTs@TiN–TiO_2_-10 are the products of CNTs@TiN@TiO_2_ being annealed in the furnace at a heating rate of 10 °C min^−1^ to 650 °C in flowing nitrogen (200 s.c.c.m).

**Recipe of CNTs@TiN (5/10/20** **nm)**

**Table:** 

	Instruction	Number	Value
1	Heater	14	150
2	Heater	15	150
3	Stabilize	15	/
4	Stabilize	14	/
5	Wait	/	7200
6	Pulse	4	0.15
7	Wait	/	20
8	Pulse	3	0.015
9	Wait	/	20
10	Goto	6	125/250/500
11	Flow	/	5

**Recipe of CNTs@TiN@TiO**_**2**_ **(2/5/10** **nm)**

**Table:** 

	Instruction	Number	Value
1	Heater	14	150
2	Heater	15	150
3	Stabilize	15	/
4	Stabilize	14	/
5	Wait	/	7200
6	Pulse	4	0.15
7	Wait	/	20
8	Pulse	3	0.015
9	Wait	/	20
10	Goto	6	125/250/500
11	Flow	/	5
12	Heater	14	150
13	Heater	15	150
14	Stabilize	15	/
15	Stabilize	14	/
16	Wait	/	600
17	Pulse	4	0.5
18	Wait	/	10
19	Pulse	0	0.03
20	Wait	/	10
21	Goto	17	40/100/200
22	Flow	/	10

### Fabrication of Li_2_S_6_ and symmetric cell assembly

The Li_2_S_6_ electrolyte was fabricated by adding Li_2_S and sulfur (molar ratio corresponds to the nominal stoichiometry of Li_2_S_6_) into the electrolyte with 1 M lithium bis(trifluoromethane sulfonyl) imide (LiTFSI) in a mixture of 1,3-dioxolane and dimethoxyethane (1:1 in volume), and then stirring at 60 °C for 24 h. The obtained Li_2_S_6_-contained electrolyte (0.5 M) with the identical anodes and cathodes of CNTs@TiN–TiO_2_-2, CNTs@TiN–TiO_2_-5, and CNTs@TiN–TiO_2_-10 were assembled into the symmetric cells for the polysulfides conversion mechanism study.

### Visual test

The electrodes of CNTs@TiN–TiO_2_-2, CNTs@TiN–TiO_2_-5, and CNTs@TiN–TiO_2_-10 were dropped into the diluted Li_2_S_6_ electrolyte (0.005 M) and kept in the argon glove box overnight.

### Fabrication of Li_2_S_8_ and Li_2_S precipitation test

Sulfur and Li_2_S in amounts of the nominal stoichiometry of Li_2_S_8_ were mixed in a tetraglyme solution at 70 °C until a dark brownish-red Li_2_S_8_ solution was formed. The cells were assembled by applying CNTs@TiN–TiO_2_-2, CNTs@TiN–TiO_2_-5, and CNTs@TiN–TiO_2_-10 as the cathodes, lithium foil as anode, and Celgard 2500 membrane as the separator. 20 μL Li_2_S_8_ (0.2 M) and blank electrolyte of Li–S batteries were added on the cathode and the anode, respectively. The cells were firstly discharged with a fixed current (0.134 mA) to 2.06 V to completely transform the Li_2_S_8_ to Li_2_S_6_, which is followed by potentiostatically discharging at 2.05 V to convert Li_2_S_6_ to Li_2_S until the current decreased to 1 × 10^−5^ mA. During the potentiostatic discharge process, time-current curves were collected to analyze the conversion from Li_2_S_4_ to Li_2_S. According to the potentiostatic discharge curves (Fig. 4), the whole discharge process was mathematically fitted into three parts representing the reduction of Li_2_S_8_ and Li_2_S_6_ and the precipitation of Li_2_S. The conversion capacity was calculated based on the areas of the precipitation of Li_2_S and the weight of sulfur in the Li_2_S_8_ electrolyte.

### Material characterization

The morphology and structure of the prepared samples were analyzed by SEM (Hitachi, S-3000N) and TEM (JEOL, JEM-ARM 200F). XRD measurements were performed with a Bruker D8 Discover diffractometer (Bruker AXS, Cu X-ray source). XPS analysis was performed using an X-ray photoelectron spectrometer (XPS-AXIS Ultra HAS, Kratos) with a monochromatic Al-Kα = 1486.6 eV X-ray source. Electrical conductivities of CNTs@TiN–TiO_2_-2, CNTs@TiN–TiO_2_-5, and CNTs@TiN–TiO_2_-10 were measured by a four-probe method on a Bruker Surface Profiler (Dektak XT). Nitrogen adsorption/desorption isotherms (77 K) were measured by the specific surface area/pore size distribution measurement instrument (BELSORP MINI X).

### Li–S battery assembly and electrochemical characterization

The obtained CNTs@TiN–TiO_2_-2, CNTs@TiN–TiO_2_-5, and CNTs@TiN–TiO_2_-10 (the average areal masses are 3.7, 3.3, and 3.1 mg cm^−^^2^, respectively) with Li_2_S_6_ electrolyte (1.2 M) were used as freestanding sulfur cathodes. Lithium metal foils and polypropylene (PP) films (Celgard 2400) were applied as anodes and separators, respectively. The electrolyte was the 1,3-dioxolane and dimethoxyethane (1:1 volume) solution containing 1 M LiTFSI and 1 wt% lithium nitrate. Coin-type (CR 2032) cells were assembled in an argon-filled glove box. The sulfur loading and the electrolyte to sulfur mass ratio were 15 mg cm^−2^ and 10 µL mg^−^^1^, respectively. The equation of Ca = Cg × Ma was used to calculate the areal capacity of coin cells, where Ca, Cg, and Ma stand for areal capacity, specific capacity, and areal sulfur loading, respectively. The gravimetric energy density of the cell can be calculated based on the equation of *E*_g_ = *f* × Cg × *V*, where *f*, Cg, and *V* stand for sulfur weight fraction of the whole device, specific capacity, and cell voltage (*V* = 2.2 V), respectively. A galvanostatic electrochemical test of the assembled cells was carried out on a Neware system in the potential range of 1.5–3.0 V at different discharge/charge current densities of 0.1–5 C. CV and EIS measurements were performed on a Metrohm Autolab electrochemical workstation. EIS curves were obtained by applying a sine wave with an amplitude of 5 mV over the frequency range from 100 kHz to 0.01 Hz.

## Supplementary information

SI

## Data Availability

All data generated or analyzed during this study are included in the published article and its Supplementary Information. The data that support the findings of this study are available from the corresponding author upon reasonable request. Source data are provided with this paper.

## Source data

Source data
