# Supplementary material for: Long-life lithium-sulfur batteries with high areal capacity based on coaxial CNTs@TiN-TiO2 sponge
Source: Nat Commun. 2021 Aug 6;12:4738. doi: 10.1038/s41467-021-24976-y (PMC8346473; doi:10.1038/s41467-021-24976-y)
Supplement: Supplementary file 1 — SI [file 41467_2021_24976_MOESM1_ESM.pdf]

Supporting Information

Long-life lithium-sulfur batteries with high areal capacity based on coaxial CNTs@TiN-TiO_2 sponge

Hui Zhang [1], Luis K. Ono [1], Guoqing Tong [1], Yuqiang Liu [1], Yabing Qi [1,*]

[1] Energy Materials and Surface Sciences Unit (EMSSU), Okinawa Institute of Science and Technology Graduate University (OIST), 1919-1 Tancha, Onna-son, Okinawa 904-0495, Japan

*Corresponding author: Yabing Qi, E-mail: Yabing.Qi@OIST.jp

List of Contents

1. Supplementary Figures

Figure S1. Morphology characterization of CNTs@TiN hybrids.

Figure S2. Electrochemical performance of CNTs@TiN hybrids at 0.2 C.

Figure S3. Morphology and electrochemical performance of CNTs@TiN@TiO_2 at 0.2 C.

Figure S5. XRD patterns of (a) CNTs@TiN (10 nm) and (b) CNTs@TiO_2 (5 nm).

Figure S5. XRD pattern of CNTs@TiN-TiO_2-5.

Figure S6. SEM image and photo of CNTs@TiN-TiO_2-5.

Figure S7. TEM images of (a) CNTs@TiN-TiO_2-2, (b) CNTs@TiN-TiO_2-5 and (c) CNTs@TiN-TiO_2-10.

Figure S8. Nitrogen adsorption/desorption isotherms of CNTs, CNTs@TiN-TiO_2-2, CNTs@TiN-TiO_2-5 and CNTs@TiN-TiO_2-10.

Figure S9. XPS spectra of CNTs@TiN-TiO_2-5 before and after lithium polysulfides adsorption.

Figure S10. CV curves of CNTs@TiN-TiO_2-2, CNTs@TiN-TiO_2-5 and CNTs@TiN-TiO_2-10 symmetric cells with and without Li_2S_6 at the scan rate of 2 mV s^{-1}.

Figure S11. Areal capacity performance of CNTs@TiN@TiO_2-5 at 0.2 C and 1 C.

Figure S12. Electrochemical performance of CNTs@TiN@TiO_2-5 at 2 C.

2. Supplementary Tables

Table S1. Electrical conductivity measurement results of CNTs@TiN hybrids by four-point

probe technique.

Table S2. Porosity comparison of CNTs, CNTs@TiN-TiO_2-2, CNTs@TiN-TiO_2-5 and CNTs@TiN-TiO_2-10.

Table S3. Performance comparison of our CNTs@TiN-TiO_2-5 with other recently reported Li-S electrodes with high areal capacity.

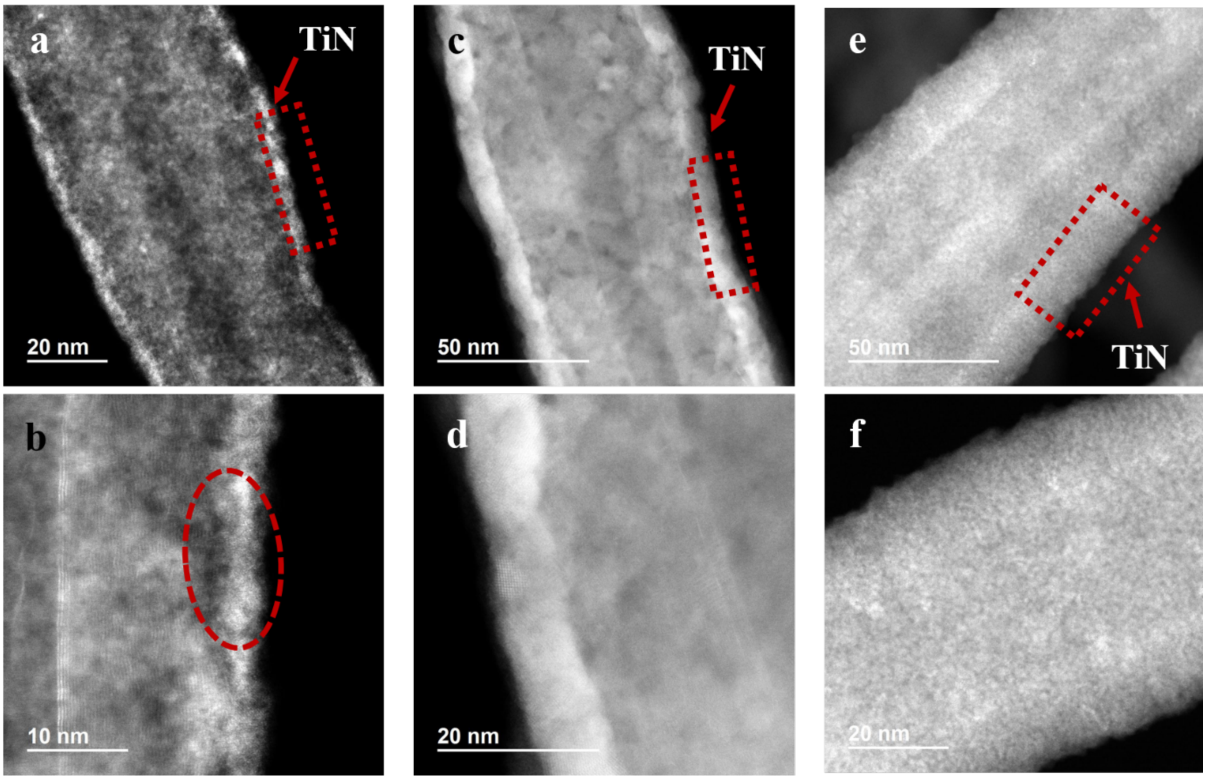

Figure S1. Morphology characterization of CNTs@TiN hybrids. TEM images of (a, b) CNTs@TiN-5, (c, d) CNTs@TiN-10, and (e, f) CNTs@TiN-20. Source data are provided as a Source Data file.

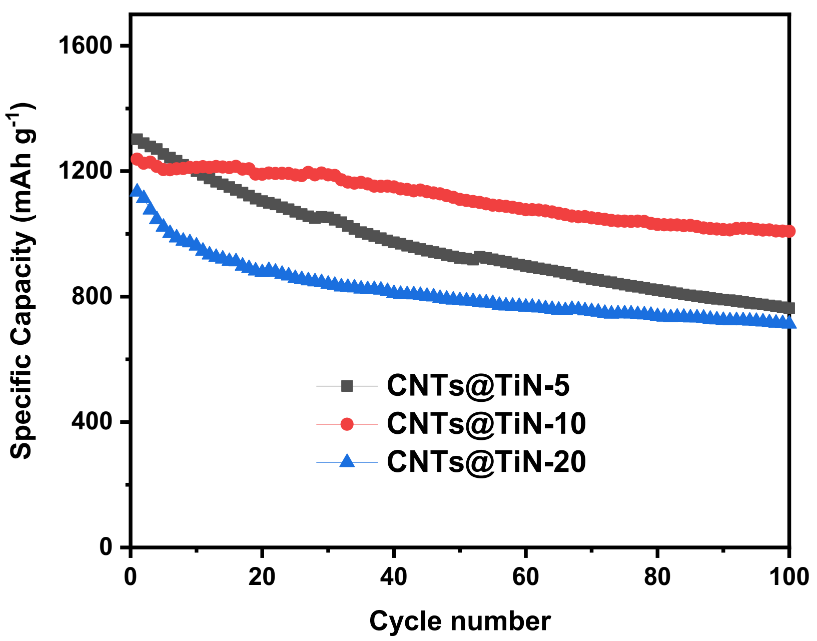

Figure S2. Electrochemical performance of CNTs@TiN hybrids at 0.2 C. Source data are provided as a Source Data file.

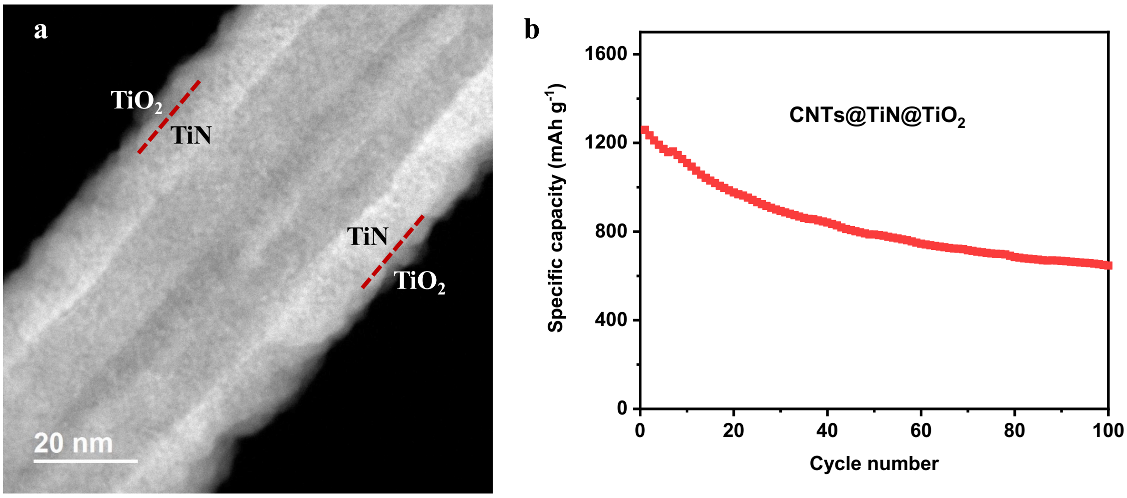

Figure S3. Morphology and electrochemical performance of CNTs@TiN@TiO_2 at 0.2 C. Source data are provided as a Source Data file.

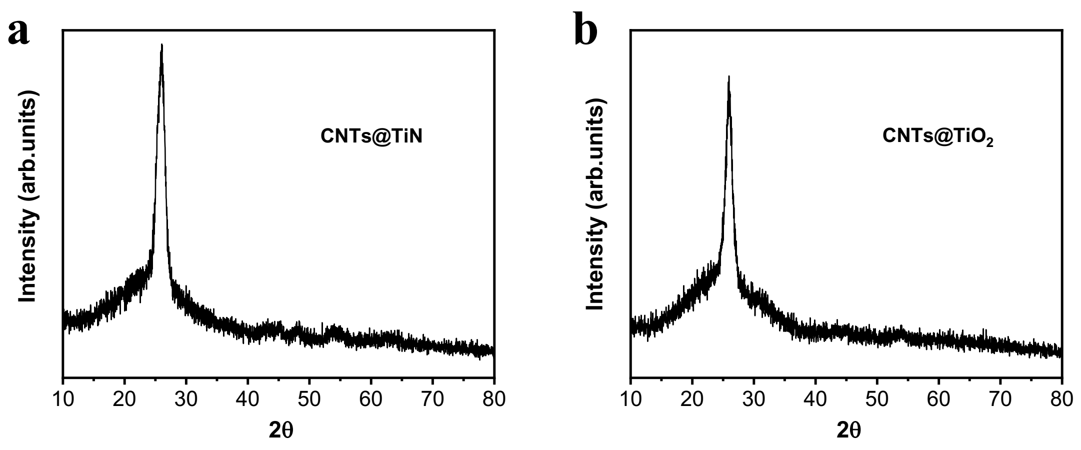

Figure S4. XRD patterns of (a) CNTs@TiN (10 nm) and (b) CNTs@TiO_2 (5 nm). Source data are provided as a Source Data file.

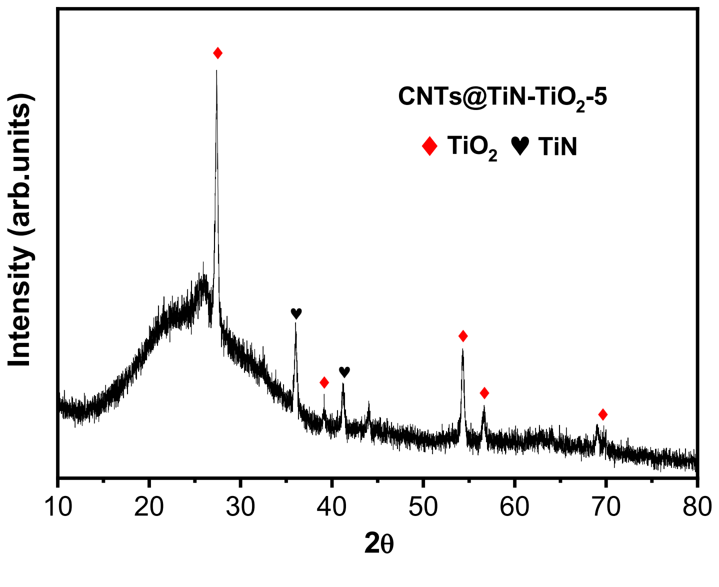

Figure S5. XRD pattern of CNTs@TiN-TiO_2-5. Source data are provided as a Source Data file.

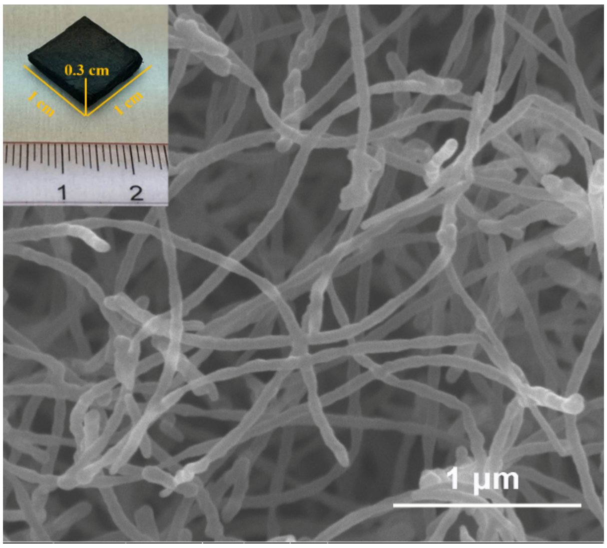

Figure S6. SEM image and photo of CNTs@TiN-TiO_2-5.

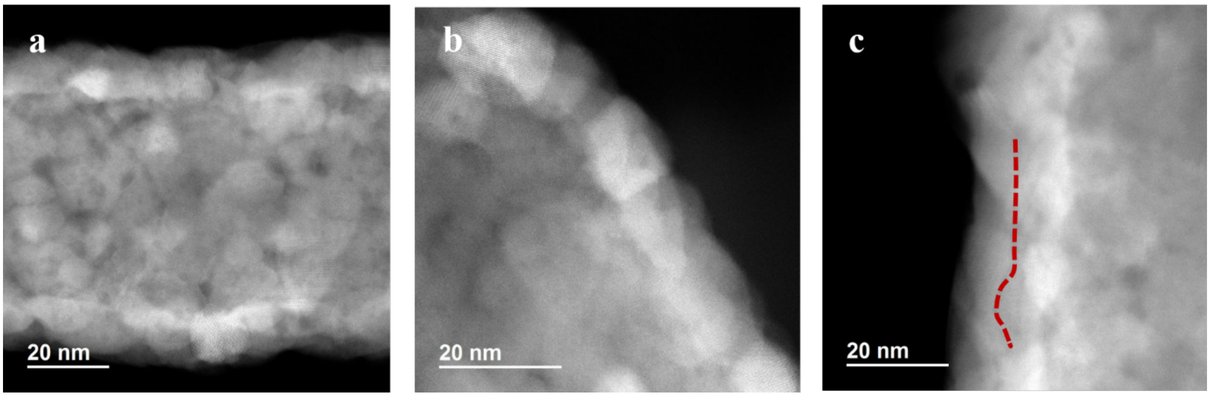

Figure S7. TEM images of (a) CNTs@TiN-TiO_2-2, (b) CNTs@TiN-TiO_2-5 and (c) CNTs@TiN-TiO_2-10. Source data are provided as a Source Data file.

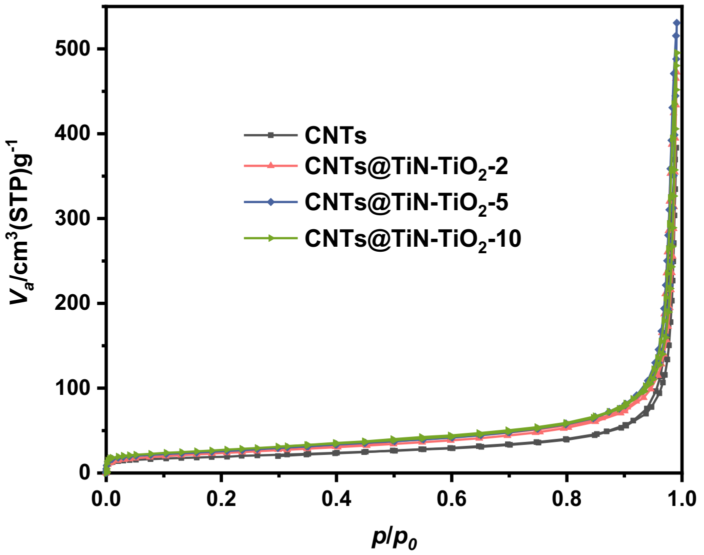

Figure S8. Nitrogen adsorption/desorption isotherms of CNTs, CNTs@TiN-TiO_2-2, CNTs@TiN-TiO_2-5 and CNTs@TiN-TiO_2-10. Source data are provided as a Source Data file.

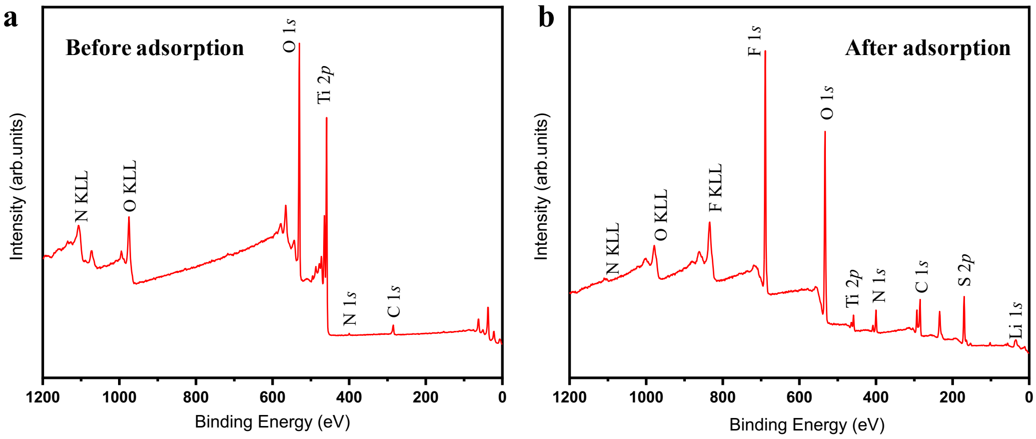

Figure S9. XPS spectra of CNTs@TiN-TiO_2-5 before and after lithium polysulfides adsorption. Source data are provided as a Source Data file.

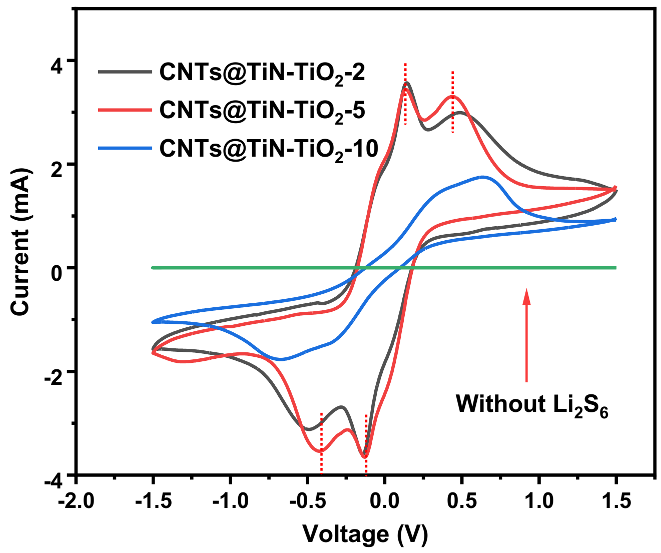

Figure S10. CV curves of CNTs@TiN-TiO_2-2, CNTs@TiN-TiO_2-5 and CNTs@TiN-TiO_2-10 symmetric cells with and without Li_2S_6 at the scan rate of 2 mV s^{-1}. Source data are provided as a Source Data file.

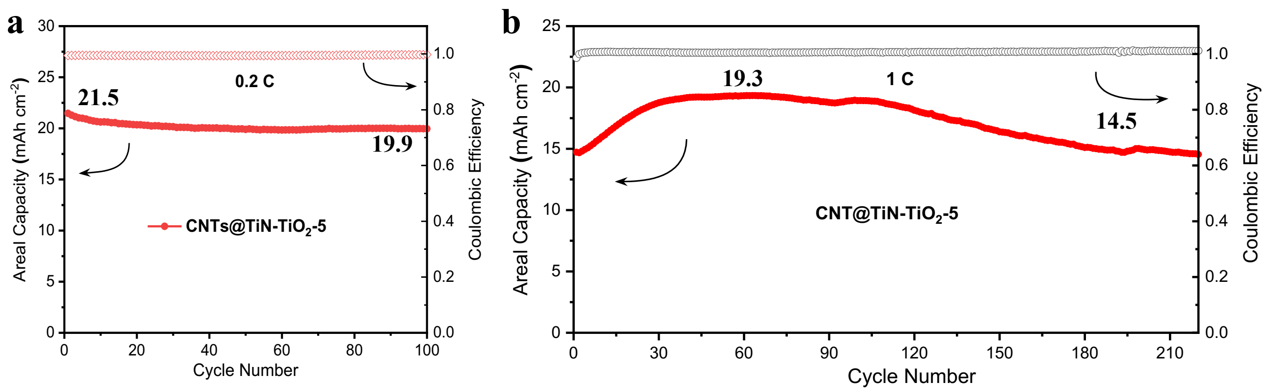

Figure S11. Areal capacity performance of CNTs@TiN@TiO_2-5 at 0.2 C and 1 C. Source data are provided as a Source Data file.

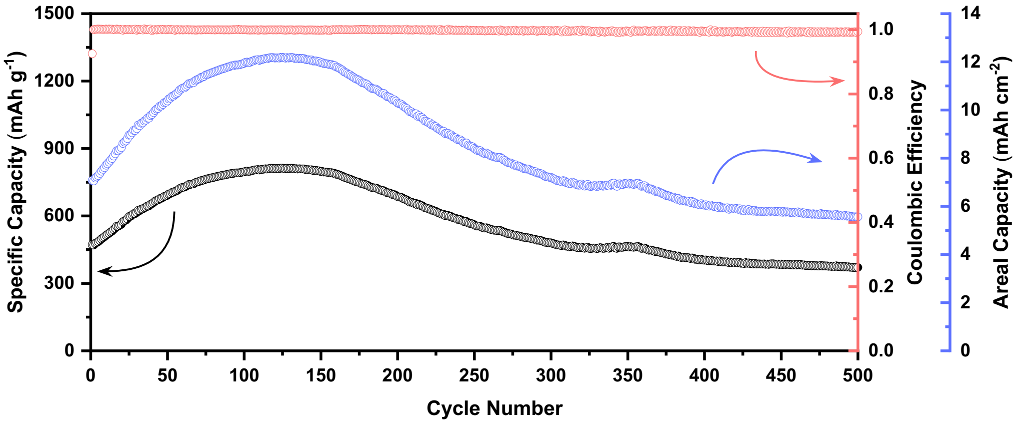

Figure S12. Electrochemical performance of CNTs@TiN@TiO_2-5 at 2 C. Source data are provided as a Source Data file.

Table S1. Electrical conductivity measurement results of CNTs@TiN hybrids by the four-point probe technique.

Sample	ρ1 (S m^{-1})	ρ2 (S m^{-1})	ρ3 (S m^{-1})	Average value (S m^{-1})
CNTs@TiN-5	3.23×10^5	3.27×10^5	3.25×10^5	$(3.25 \pm 0.02)\times10^5$
CNTs@TiN-10	2.87×10^5	2.65×10^5	2.67×10^5	$(2.73 \pm 0.10)\times10^5$
CNTs@TiN-20	9.87×10^4	9.58×10^4	9.64×10^4	$(9.70 \pm 0.13)\times10^4$

Table S2. Porosity comparison of CNTs, CNTs@TiN-TiO_2-2, CNTs@TiN-TiO_2-5 and CNTs@TiN-TiO_2-10.

	Specific surface area (m^2 g^{-1})	Total mesopore volume (cm^3 g^{-1})	Average mesopore diameter (nm)
CNTs	67.098	0.5898	35.179
CNTs@TiN-TiO_2-2	84.616	0.6901	32.620
CNTs@TiN-TiO_2-5	90.980	0.7614	33.474
CNTs@TiN-TiO_2-10	96.133	0.7304	30.391

Table S3. Performance comparison of our CNTs@TiN-TiO_2-5 with other recently reported Li-S electrodes with high areal capacity.

**Sulfur loading/ (mg cm^{-2})**	**Areal capacity (mAh cm^{-2})**		**Areal capacity (mAh cm^{-2})/ n^{th} cycles/C-rate**	**E/S (μL mg^{-1})**	**Li_2S_6 (M)**			**Ref.**
	**Max**	**100 cycles**			**Adsorption**	**Symmetric cells**	**Li-S cells**	
Sulfur-based								
9.8	10.3	6.86	6.3/350/0.2	7.34				7
21.2	23.3	17.04	14.8/150/0.1	3.53				13
10.2	9.26	-	8.18/70/0.2	4.4	0.0048	0.1		26
19.1	19.3	9	9/100/0.1	NA				27
12	13.5	-	10.8/50/0.03	20				28
4	3.60	2.90	2.9/100/0.1	15				32
3.7	3.40	3.25	2.6/500/0.5	10		0.2		33
5.4	4.00	3.00	3/100/0.02	n/a				35
1.5	2.09	1.29	0.82/300/0.2	30				36
Polysulfides-based								
9.6	12	-	9.96/60/0.5	10	0.05		0.52	23
12	11.4	9.73	7.3/200/0.1	6			1	25
57.6	38.57	33.68	28.39/200/0.1	4.2			1.5	29
61.4	57.6	-	42.9/50/0.1	6.8			1	30
5	6.56	4.77	4.13/160/0.2	NA	0.002		0.5	31
18.1	20.00	-	12/70/0.2	5.66			1	34
**15**	**21.5**	**19.9**	**19.9/100/0.2**	**10**	**0.005**	**0.5**	**1.2**	**Our work**
	**19.8**	**14.5**	**14.5/220/1**					
